# Acute transverse myelitis arising after combined general and thoracic epidural anesthesia

**DOI:** 10.1186/s40981-015-0006-5

**Published:** 2015-08-27

**Authors:** Tetsuya Shimada, Shinya Yufune, Motoshi Tanaka, Ryosuke Akai, Yasushi Satoh, Tomiei Kazama

**Affiliations:** Department of Anesthesiology, National Defense Medical College, 3-2 Namiki, Tokorozawa, Saitama 359-8513 Japan

**Keywords:** Acute transverse myelitis, Paralysis, Epidural anesthesia

## Abstract

Acute transverse myelitis after surgery is a rare condition, but this complication is devastating. The relationship between anesthetic procedures and acute transverse myelitis is controversial. A 46-year-old woman was scheduled a colostomy closure, and general anesthesia with thoracic epidural anesthesia was performed. Epidural catheter was inserted at the T10–11 interspace, and insertion was smooth, and no blood or cerebrospinal fluid leakage was seen. However, 28 h after the surgery, the patient complained motor, sensory, and autonomic dysfunction. Two days after onset, a magnetic resonance imaging study demonstrated intramedullary hyperintensity, particularly in the gray matter, extending from T5–T9 and then diagnosed with acute transverse myelitis followed by the several examinations. High-dose IV methylprednisolone treatment was initiated and neurologic function restored 2 months after onset. Transverse myelitis may unpredictably occur following surgery. We are not able to determine the pathogenic relationship between anesthesia and myelitis with certainty, but proper diagnostic approach to myelitis may improve the prognosis.

## Background

Severe neurologic complications following postoperative epidural analgesia occur infrequently or rarely with a reported prevalence of only 0.005–0.070 % [1]. Most recognized are epidural hematoma or abscess, whereas no systematic reviews, which show the relationship between anesthetic procedures or medications and acute transverse myelitis, have been published, and only few cases of acute myelitis after thoracic epidural anesthesia have been reported [2, 3]. In this report, we present a patient who postoperatively developed acute myelitis with an epidural catheter in situ. The patient subsequently developed a complication of treatment with steroids of the myelitis.

## Case presentation

A 46-year-old woman (53.6 kg, 152 cm tall, and ASA PS II) was scheduled for a colostomy closure. She gave written informed consent and approved the reporting of her case. Thirteen months prior to colostomy closure, she had been treated for generalized peritonitis following Hartmann’s operation for perforation of the sigmoid colon cause by colorectal cancer. Hartmann’s operation was under combination of general and epidural anesthesia. And she received general anesthesia without epidural for her generalized peritonitis. Both of their anesthetic procedures were uneventful. The patient was receiving outpatient chemotherapy. Right after finishing the treatment, the patient was planned to administer to have colostomy closure surgery. She also had a history of asthma, which was treated with inhaled budesonide. She had no other past medical history. At presentation, she was not taking any herbal preparations or anticoagulants and there were no contradictions for epidural anesthesia.

Epidural catheterization was performed in the right lateral position. After skin preparation with 0.5 % chlorhexidine, epidural space was identified by loss of resistance technique with normal saline using a 17-gauge Tuohy needle at the T10–11 interspace, and a 20-gauge epidural catheter (Perifix® Soft Tip catheter) was threaded 5.5 cm and taped securely. Catheter insertion was smooth, and no blood or cerebrospinal fluid leakage was seen. The conduct of the epidural insertion was unremarkable, and following a test dose, there was no evidence of subarachnoid block.

General anesthesia was induced by target-controlled infusion (TCI) of propofol at a target plasma concentration of 3 μg/ml. Rocuronium (0.7 mg/kg) was administered after loss of response to verbal commands. Remifentanil was initiated at a rate of 0.3 μg/kg/min, and propofol was adjusted to maintain a BIS index of 40–60. Ten minutes before skin incision, 6 ml of 0.2 % ropivacaine and 100 μg of fentanyl were administered through the epidural catheter. A mobile disposable negative-pressure infusion pump (Coopdech Syrinjector, Daiken Medical, Osaka, Japan) primed with a total volume of 120 ml anesthetic (113 ml of 0.2 % ropivacaine, 300 μg of fentanyl, and 2.5 mg of droperidol) was used for continuous epidural infusion at a rate of 5 ml/h starting 10 min after skin incision. The surgical procedure itself was uneventful, and recovery was satisfactory. The patient reported no pain at the surgical site, and no lower extremity paralysis was observed immediately after the operation.

**Fig. 1:**
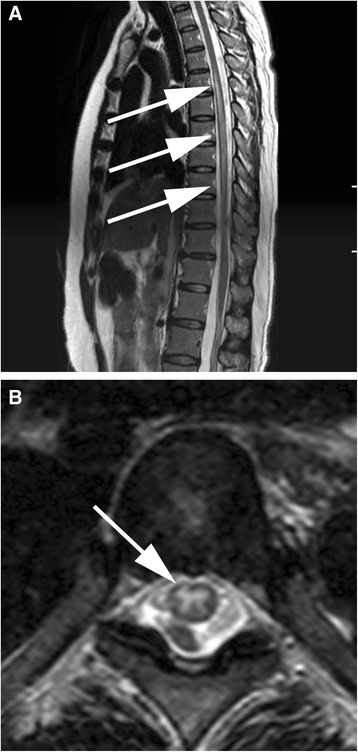
Intramedullary hyperintensity, particularly in the gray matter, extending from T5–T9 levels on T2-weighted magnetic resonance imaging (*white arrows*). **a** Sagittal view. **b** Axial view

However, 28 h after entering the PACU, she developed paralysis in her right lower extremity and paresthesia in her right trunk, and epidural continuous infusion was stopped. However, symptoms remained 12 h after onset, and the epidural catheter was removed after confirming no sign of coagulopathy. Dysuria appeared 17 h after onset. Examination by a neurologist revealed paraparesis (impaired lower extremity movement, particularly in her right leg), decreased thermal nociception on the right dermatome T6–12, hyperesthesia below the right dermatome T12, and bladder and rectal dysfunction. Two days after onset, an emergent thoracolumbar spine magnetic resonance imaging (MRI; 1.5T system, Ingenia, Philips Medical Systems, Best, The Netherlands) demonstrated intramedullary hyperintensity, particularly in the gray matter, extending from T5–T9 on T2-weighted and diffusion-weighted MR images (Fig. 1). No space-occupying lesion, such as hematoma or abscess, and no intracranial lesion, such as hemorrhage or infarction, were seen. Due to her history of asthma, contrast-enhanced MRI was not performed. The following day, the lumbar puncture was performed after confirming no sign of coagulopathy, and inflammation within the spinal cord was indicated by celebrospinal fluid (CSF) pleocytosis and elevated IgG index, but no viral infection was detected. The drug-induced lymphocyte stimulation test for ropivacaine showed no allergic history. On the basis of the above results, she was diagnosed with acute transverse myelitis.

High-dose IV methylprednisolone treatment was initiated (first treatment: 1 g daily for 3 days). Paresthesia on the right side and bladder and rectal disturbances slightly recovered, and she began to walk using a standard pick-up walker. Additional high-dose IV methylprednisolone treatments (second: 1 g daily for 3 days and third: 500 mg daily for 3 days) resulted in gradual improvement of sensorimotor symptoms. On the other hand, D-dimer concentration was elevated from 6.2 μg/ml before the first treatment to 13.7 μg/ml after the second treatment. After the second treatment, no lower limb deep venous thrombosis was observed by color Doppler ultrasound imaging of the lower extremity. After the third treatment, however, dyspnea occurred and her D-dimer concentration was elevated to 31.1 μg/ml, and she was examined by pulmonary scintigraphy, which showed ventilation-perfusion mismatching in both lungs (right posterior, right superior, right anterior-basal, right lateral-basal, right posterior-basal, and left lateral-basal segments), indicative of multiple pulmonary embolisms. Heparin therapy to maintain activated partial thromboplastin time with the therapeutic target range of 50–70 s was initiated. Two days after initiating heparin treatment, symptoms of dyspnea disappeared. Following establishment of a therapeutic prothrombin time/international normalized ratio with warfarin, the heparin was ceased.

Forty-seven days after onset, paraparesis of the right lower extremity had recovered. Results of manual muscle tests (MMTs) were 3/5 for iliopsoas, 3/5 for hamstrings, 4/5 for quadriceps, and 5/5 for other muscles, and she could walk with a medical cane. Forty-nine days after onset, thermal nociception and hyperesthesia had diminished (although incompatibility still existed) and bladder and rectal disturbance had recovered. Further, she was independent in defecation. Fifty-one days after onset, results of MMTs were 5/5 for iliopsoas and hamstrings; furthermore, she could walk without a cane.

### Discussion

Transverse myelitis is, rare with only 1–8 cases per million per year reported [4], a focal inflammatory disorder of the spinal cord, resulting in motor, sensory, and autonomic dysfunction. Longitudinal case series of acute transverse myelitis reveal that approximately one third of patients recover with little to no sequelae, one third are left with moderate degree of permanent disability, and one third have severe disabilities [5]. Transverse Myelitis Consortium Working Group proposed diagnostic criteria, as well as a diagnostic approach algorism when we see patients with neurologic deficits corresponding to a spinal disorder [6].

According to the algorism, the first priority is to exclude an extra axial compressive etiology. The working group suggests to conduct gadolinium-enhanced MRI within 4 h after recognizing the deficits to rule out the compressive etiology like hematoma or abscess. These complications were highly possible in this case; therefore, we should have performed MRI much earlier, though in this specific patient with a history of asthma, possible anaphylactoid reaction precluded the use of contrast agents [7].

The second priority is to define the presence or absence of spinal cord inflammation. Non-inflammatory causes of myelopathy include ischemia, radiation, epidural lipomatosis, or fibrocartilagious embolism. In this case, we confirmed CSF pleocytosis and elevated IgG index, so we considered some inflammatory reaction as its pathogenesis.

The third priority is to define the extent of demyelination by using brain contrast MRI and visual evoked potential. If myelination of the brain or the brain with optic nerve is detected, multiple sclerosis, acute disseminated encephalomyelitis, or disease-associated acute transverse myelitis are possible diagnosis. Myelination of the optic nerve or tract may indicate neuromyelitis optica. If only the spinal cord was demyelinated, the diagnosis is acute transverse myelitis due to idiopathic or disease-associated etiology. We could not use a contrast medium because of the reason mentioned above, but by checking non-contrast MRI and visual evoked potential (VEP; delayed VEP was observed on 43 days after onset), we have concluded that this patient had acute transverse myelitis with some reasons.

Once diagnosis of transverse myelitis is established, it is useful to clarify the cause to decide on therapeutic strategy and assess prognosis. However, even after several years of follow-up, 15–36 % of transverse myelitis patients cannot be given a more specific diagnosis [4].

What is possible causes inducing the acute transverse myelitis in this patient? The relationship between anesthesia and acute myelitis is controversial, with only a few published studies addressing a possible association.

We speculate mainly two reasons to cause acute transverse myelitis in this patient. One of the reasons is the neurotoxicity derived from local anesthetics. It is reported that histologic damage in the nerve roots and the spinal cord was less severe after injection with epidural local anesthetics than with intrathecal local anesthetics in rats [8]. And it was also reported that the inadvertent intrathecal administration of intended epidural local anesthetic can occur in a clinical situation [9]. Considering these reports and the devastating spinal cord injury in this case, we speculate that the tip of the epidural catheter could exist in the intrathecal not in the epidural space; as a result, the local anesthetic continued to affect the spinal cord and induced the devastating damage.

The other predicted pathogenesis is an autoimmune reaction induced by chemical stimulation like local anesthetic or mechanical stimulation by a catheter or an epidural needle, which in turn caused acute transverse myelitis. According to the algorithm, autoimmune disease like multiple sclerosis, acute disseminated encephalomyelopathy, or neuromyelitis optica must be considered as a differential diagnosis.

One of the reason why we would speculate the cause as an autoimmune reaction is that elevated IL-6 was detected in the CSF (179 pg/ml, normal <4.0 pg/ml) and serum (5.82 pg/ml, normal <4.0 pg/ml) in this patient. Additionally, a delayed VEP may suggest the existence of potential autoimmune disease such as neuromyelitis optica. A case with neuromyelitis optica arising after a spinal anesthesia with bupivacaine was reported [10]. Originally, the patient might have autoimmune disease potentially, and it might become apparent after the surgery by the trigger of anesthesia or surgery itself, or it might be merely an innocent by-stander. Fortunately, she could have recovered with little sequelae; however, we cannot deny with certainty her remission of autoimmune reaction by unknown etiology. Further continuous observation by neurologist should be necessary in this case.

Interestingly, Gutowski and Davies published a case with acute transverse myelitis after general anesthesia without epidural anesthesia [11], and this case shows that transverse myelitis may arise coincidentally after the surgery in which no local anesthetic procedure has been used. This case arises some caution to anesthesiologists, that epidural or spinal anesthesia may be just innocent by-standers even when we see patients with some neurologic disorders after epidural or spinal procedures. Despite the absence of evidence, administration of high-dose IV methylprednisolone is the first treatment to hasten recovery, reduce activity, and restore neurologic function [4].

In addition to acute transverse myelitis, we have also faced with multiple pulmonary embolisms in this case. Known etiologic factors predisposing to venous thromboembolism include reduced mobility, malignant disease, and history of venous thromboembolism [12]. Cancer patients are considered at a significantly increased risk of developing venous thromboembolism, particularly while receiving systemic chemotherapy [13]. Female sex is another reported predisposing factor for thrombosis [12]. The patient in this case was a woman with a history of colorectal cancer who had received outpatient chemotherapy and reduced mobility due to transverse myelitis. In addition, intermittent pneumatic leg compression was performed, but pharmacological prophylaxis was not performed in this case. Combined prophylaxis could prevent from developing pulmonary embolisms [14].

## Conclusions

In conclusion, transverse myelitis may unpredictably occur following surgery. While we are not able to determine the pathogenic relationship between anesthesia and myelitis with certainty, this complication is devastating. Despite we may not be able to prevent similar events, proper diagnostic approach to myelitis may improve the prognosis.

## Consent

Written informed consent was obtained from the patient for publication of this case report and any accompanying images. A copy of the written consent is available for review by the Editor-in-chief of this journal.

## Abbreviations

ATM: acute transverse myelitis
TCI: target-controlled infusion
BIS: bispectral index
MRI: magnetic resonance imaging
CSF: celebrospinal fluid
MMTs: manual muscle tests
VEP: visual evoked potential
